# Supplementary material for: Conditional chemoconnectomics (cCCTomics) as a strategy for efficient and conditional targeting of chemical transmission
Source: eLife. 2024 Apr 30;12:RP91927. doi: 10.7554/eLife.91927 (PMC11060718; doi:10.7554/eLife.91927)
Supplement: Figure 3—source data 3. [file elife-91927-fig3-data3.zip › Figure 3 source data3 part 2.zip!/Figure 3-Source data 3-part2/1002-9-CRISPResso_Report_e53dd8m5.zip!/CRISPResso_on_1002_10/2a.Nucleotide_percentage_quilt.pdf]

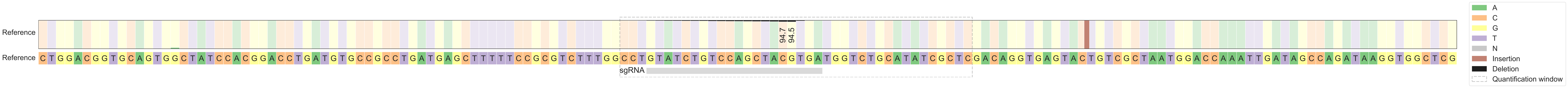

Reference
94.7
94.5
Reference
CTGGACGGTGCAGTGGCTATCCACGGACCTGATGTGCCGCCTGATGAGCTTTTTCCGCGTCTTTGGCCTGTATCTGTCCAGCTACGTGATGGTCTGCATATCGCTCGACAGGTGAGTACTGTCGCTAATGGACCAAATTGATAGCCAGATAAGGTGGCTCG
sgRNA
A
C
G
T
N
Insertion
Deletion
Quantification window
